# Detection of *Campylobacter* spp. in water by dead‐end ultrafiltration and application at farm level

**DOI:** 10.1111/jam.14379

**Published:** 2019-07-22

**Authors:** S. Ferrari, S. Frosth, L. Svensson, L.‐L. Fernström, H. Skarin, I. Hansson

**Affiliations:** ^1^ Department of Microbiology National Veterinary Institute Uppsala Sweden; ^2^ Department of Biomedical Sciences and Veterinary Public Health, Faculty of Veterinary Medicine and Animal Science Swedish University of Agricultural Sciences Uppsala Sweden; ^3^ Department of Disease Control and Epidemiology National Veterinary Institute Uppsala Sweden

**Keywords:** broiler chickens, *Campylobacter*, dead‐end ultrafiltration, DEUF, spiked water, water

## Abstract

**Aims:**

The purposes were to evaluate the detection of low levels of *Campylobacter* in water by dead‐end ultrafiltration (DEUF) to determine the sensitivity and suitability for use under field condition.

**Methods and Results:**

The DEUF technique followed by detection according to ISO 10272 was tested on artificially and naturally contaminated water. *Campylobacter* were detected in all samples spiked with more than 10 CFU 60 l^−1^ and in four of nine samples with a concentration below 10 CFU 60 l^−1^ water. Naturally contaminated water from five different broiler producers was analysed. *Campylobacter* were detected in four of 12 samples from ponds near the houses and in three of 24 samples from water pipes inside the broiler houses, but not in tap water sampled at the entrance of the broiler houses.

**Conclusions:**

The results indicate that DEUF is useful for detection of low numbers of *Campylobacter* in large volumes of water.

**Significance and Impact of the Study:**

Contaminated water is an important source for transmission of *Campylobacter* to broilers and humans. The concentration of *Campylobacter* is usually low with a high level of background microbiota. This study shows the advantages of DEUF both in the laboratory and under field conditions.

## Introduction

*Campylobacter* infection in humans (campylobacteriosis) is the most frequently reported zoonotic disease in many parts of the world (EFSA and ECDC, 2018b; NNDSS, 2019). The Centers for Disease Control and Prevention (CDC) estimates that >1·3 million people in the United States contract campylobacteriosis every year (FoodNet, 2017), while according to a summary report from the European Food Safety Authority (EFSA) and European Centre for Disease Prevention and Control (ECDC), approximately 250 000 human cases per year were reported in Europe in 2016 and 2017 (EFSA and ECDC, 2018b). The main sources of human campylobacteriosis are broiler chickens (*Gallus gallus domesticus*) and broiler products. However, other risk factors exist, for instance unpasteurized milk and contaminated drinking water (Kuhn *et al.,* 2017; EFSA and ECDC, 2018a). Most human cases of campylobacteriosis are sporadic, but wider outbreaks can occur, often linked to water and unpasteurized milk (Taylor *et al.,* 2013; FoodNet, 2017; EFSA and ECDC, 2018b)*. Campylobacter* spp. are common in natural waters, such as streams, rivers and lakes, due to discharge from wastewater treatment plants, runoff from pastures after rain and direct contamination with faeces from infected animals or humans (Jones, 2001; Diergaardt *et al.,* 2004; Pitkanen, 2013; Guzman‐Herrador *et al.,* 2015; Moreira and Bondelind, 2017). Waterborne outbreaks caused by *Campylobacter* are reported especially in countries where groundwater sources commonly used as drinking water supply are not chlorinated (Hanninen *et al.,* 2003; Guzman‐Herrador *et al.,* 2015; Kuhn *et al.,* 2017; Moreira and Bondelind, 2017). In New Zealand, consumption of untreated water was the third most reported risk factor associated with campylobacteriosis in 2015 (ESR, 2016). *Campylobacter* can survive in water for up to several months, depending on environmental conditions and on the strain (Rollins and Colwell, 1986; Korhonen and Martikainen, 1991; Chan *et al.,* 2001; Obiri‐Danso *et al.,* 2001; Cools *et al.,* 2003; Trigui *et al.,* 2015; Nilsson *et al.,* 2018). An important survival strategy for *Campylobacter* is to form or integrate into biofilms, which enables the micro‐organism to survive in environments where it would normally perish. An Irish study on broiler chicken houses reported difficulties with efficient cleaning, with *Campylobacter* being isolated in samples from water pipes even after disinfection (Battersby *et al.,* 2017). This suggests that biofilms containing *Campylobacter* that form within water pipes in broiler chicken houses pose a risk of broiler chicken flocks being colonized with *Campylobacter* (Teh *et al.,* 2016).

The concentration of *Campylobacter* in contaminated drinking water is usually low, less than 10 colony‐forming units per litre (CFU l^−1^) (Savill *et al.,* 2001; Diergaardt *et al.,* 2004; Miller and Mandrell, 2005; St‐Pierre *et al.,* 2009; Banting *et al.,* 2016). However, due to the low infectious dose of *Campylobacter*, which is experimentally determined to be 500 CFU (Robinson, 1981) or 800 CFU per person (Black *et al.,* 1988), even low concentrations of *Campylobacter* in water can pose a health risk. For this reason, sensitive and reliable detection methods for *Campylobacter* in water are of great importance. Existing reference methods are limited by the volume of water that can be processed. The methods described in ISO 17995:2005 (Water quality—Detection and enumeration of thermotolerant *Campylobacter* species) (International Organization for Standardization (ISO) 2017) and NMKL 119:2007 (Thermotolerant *Campylobacter*—Detection, semi‐quantitative and quantitative determination in foods and drinking water) (NMKL, 2007) require three different volumes (10, 100, and 1000 ml) of the same water sample to be analysed by membrane filtration. Analysis of larger sample volumes increases the chances of detecting *Campylobacter.* When the volume was increased from 1 to 3 l water in a Finnish study using membrane filtration, the number of samples where *Campylobacter* was detected increased from three to five samples out of 20 (Hanninen *et al.,* 2003). In three waterborne outbreaks caused by *C. jejuni* in Finland, multiple sampling and analysis were performed on both small and large volumes of water (4–20 l) and the results showed that analysis of multiple samples and large sample volumes improved *Campylobacter* detection rates (Hanninen *et al.,* 2003).

Dead‐end ultrafiltration (DEUF) is an alternative when analysing water samples, as it is possible to process large volumes of water and simultaneously concentrate bacteria, viruses and protozoa based on size exclusion (Hill *et al.,* 2007; Smith and Hill, 2009). Compared with transporting large volumes of water to the laboratory for analysis, use of the DEUF technique under field conditions decreases the costs and labour required for the analysis. The DEUF technique is simple and portable for field use and does not require any special laboratory training for field personnel. Water from different sources, such as surface water, rainwater and drinking water for animals and humans, can be filtered on‐site. Instead of bulk water samples, the filters are then transported to the laboratory for final processing and analysis. Ultrafiltration has previously been tested in different studies. For example, in an Australian analysis of household tap water from rainwater tanks, samples of approximately 19 l from each rainwater tank and household taps were concentrated to approximately 100 ml, *Campylobacter* were detected in 21% of samples (Ahmed *et al.,* 2012). In another Australian study in which 20 l of urban storm water was concentrated using hollow fibre ultrafiltration to 100 ml, and then further concentrated to 10 ml, *Campylobacter* spp. were detected in all samples by PCR analysis and *C. jejuni* was found in 17 out of 22 samples (Sidhu *et al.,* 2012).

The aim of the present study was to evaluate the DEUF technique for detection of low concentrations of *Campylobacter* in artificially contaminated water samples, in order to determine the sensitivity of the method. A second aim was to evaluate the suitability of DEUF for detection of *Campylobacter* in naturally contaminated water from different sources at the farm level.

## Materials and methods

### Dead‐end ultrafiltration

**Figure 1:**
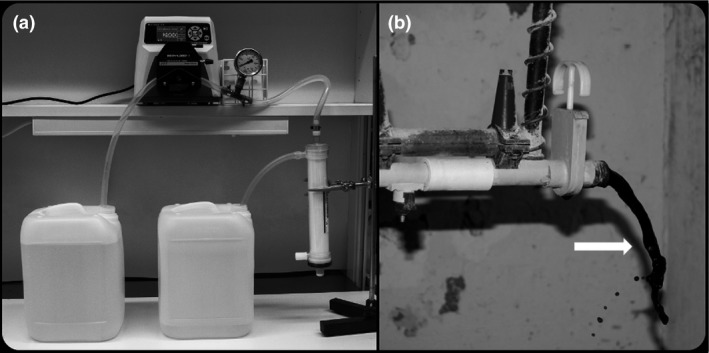
(a) Set‐up used for filtration of artificially contaminated water by dead‐end ultrafiltration at the laboratory. (b) Sampling heavily discoloured water (arrow) from the water pipes rinsed by increasing and decreasing the pressure of water and air.

Large water samples (60 l each) were concentrated by DEUF using REXEED‐25A (Scandinavian Medical Sweden AB, Knivsta, Sweden) as previously described by Hill et al. (2007). In brief, each sample was pumped with a Cole‐Parmer Masterflex L/S peristaltic pump (model 7528‐10) (Vernon Hills, IL, USA) with sterile high‐performance, platinum‐cured L/S 36 silicone tubing (Masterflex). Autoclaved tubes and connectors were used in each analysis. To minimize adsorption of the bacteria, the hollow fibre filter REXEED‐25A was blocked with 5% sterile calf serum (P30‐1985; PAN Biotech, Wimborne, UK) by entering one end of the fibres and exiting the other end and then recirculating to the fibre entrance without the solution crossing the fibre wall for 20 min at a pump rate of 425 ml min^−1^. Thereafter, the filters were sealed in a bag and stored at 4°C until use. The calf serum was rinsed from the ultrafilter prior to each experiment using 1 l Super‐Q water (381260; SVA, Uppsala, Sweden). The ultrafilters were set up with the input port on the top, connected with L/S 36 tubing to an oil‐filled pressure gauge and clamped with plastic tape to prevent leakage. The pressure gauge was then fastened with L/S 36 tubing, which extended into the pump head and to a plastic bucket of water (Fig. 1).

### Artificial contamination of water

Seven different *Campylobacter* strains of different multi‐locus sequence types were used for artificial contamination of water (Table 1). Two strains, *C. jejuni* CCUG11284 and *C. coli* CCUG11283, were reference strains and one, *C. jejuni* 18C94, was a strain isolated from a caecum sample from broiler chickens collected within the Swedish *Campylobacter* monitoring programme for broiler chickens (Hansson *et al.,* 2007). The remaining four strains, CA27, CA79, CA296, CA358, were isolated from naturally contaminated water at farm level (Table 1). One of the strains, CA27, was isolated from water from a water pipe in a broiler chicken house and the other three strains were isolated from water samples from a pond close to a broiler chicken farm (Table 2).

**Table 1:** *Campylobacter* detection by dead‐end ultrafiltration in 60‐l samples of water artificially contaminated with different concentrations of *Campylobacter* spp.

Species	Strain	ST	Concentration (CFU 60 l^−1^)				
*C. jejuni*	CCUG11284	ST‐403	–	1–10*	10–100*	100–1000*	–
*C. jejuni*	CCUG11284	ST‐403	0–1	1–10*	10–100*	–	–
*C. jejuni*	CCUG11284	ST‐403	0–1	1–10	10–100*	–	–
*C. jejuni*	CCUG11284	ST‐403	0–1	1–10	10–100*	–	–
*C. jejuni*	18C94	ST‐7516	0–1	1–10*	10–100*	–	–
*C. coli*	CCUG 11283	ST‐900	–	1–10	10–100*	100–1000*	–
*C. jejuni*	CA27	ST‐257	–	1–10	10–100*	100–1000*	–
*C. jejuni*	CA79d	ST‐583	–	–	10–100*	100–1000*	1000–2000*
*C. jejuni*	CA296_1_3	ST‐9198	–	1–10	10–100*	100–1000*	–
*C. jejuni*	CA358	ST‐7809	–	1–10*	10–100*	100–1000*	–

–: not done.

* Spiking levels at which *Campylobacter* spp. were detected.

**Table 2:** Results of the PODLOD calculations based on the data for all strains used in the artificial contaminated water

Matrix		Matrix effect *F_i_*	Log matrix effect *f_i_*	SD of log matrix effect *S_fi_*	LOD_50%_ = 50% limit of detection in cfu/ml			LOD_95%_ = 95% limit of detection in cfu/ml			Test statistic matrix effect │*Z_i_*│
No.*i*	Designation matrix*_i_*				Detection limit *d*_0·5,_ *_i_*	Lower conf. limit *d*_0·5,_ *_i,L_*	Upper conf. limit *d*_0·5,_ *_i,U_*	Detection limit *d*_0·95,_ *_i_*	Lower conf. limit *d*_0·95,_ *_i,L_*	Upper conf. limit *d*_0·95,i_ *_,U_*	
1	*C. jejuni* CCUG11284	∞	∞	Because every inoculated tube is positive							
2	*C. jejuni* CCUG11284	0·596	−0·517	1·127	1·9E‐05	2·0E‐06	1·8E‐04	8·4E‐05	8·8E‐06	8·0E‐04	0·408
3	*C. jejuni* CCUG11284	0·117	−2·148	1·107	9·9E‐05	1·1E‐05	9·1E‐04	4·3E‐04	8·8E‐06	0·004	1·929
4	*C. jejuni* CCUG11284	0·227	−1·484	1·107	5·1E‐05	5·6E‐06	4·7E‐04	2·2E‐04	8·8E‐06	0·002	1·231
5	*C. jejuni* 18‐C94	0·285	−1·254	1·127	4·0E‐05	4·2E‐06	3·9E‐04	1·7E‐04	8·8E‐06	0·002	0·931
6	*C. coli* CCUG11283	0·077	−2·566	1·127	1·5E‐04	1·6E‐05	0·001	6·5E‐04	8·8E‐06	0·006	1·721
7	*C. jejuni* CA27	0·055	−2·905	1·127	2·1E‐04	2·2E‐05	0·002	9·1E‐04	8·8E‐06	0·009	1·433
8	*C. jejuni* CA79	∞	∞	Because every inoculated tube is positive							
9	*C. jejuni* CA296	0·095	−2·352	1·127	1·2E‐04	1·3E‐05	0·001	5·2E‐04	5·5E‐05	0·005	1·753
10	*C. jejuni* CA358	∞	∞	Because every inoculated tube is positive							
Combined results		0·200	−1·611	0·397	5·8E‐05	2·6E‐05	1·3E‐04	2·5E‐04	1·1E‐04	5·5E‐04	3·366
		Based on the data of matrices 1, 2, 3, 4, 5, 6, 7, 8, 9 and 10									

The strains were cultured in 5 ml Brain Heart Infusion (Oxoid CM1135, Basingstoke, UK) broth and incubated for 24 ± 2 h at 41·5 ± 1°C in a microaerophilic atmosphere generated using the Anoxomat system (Mart BV, Lichtenvoorde, the Netherlands). A 10‐fold serial dilution, 10^−1^–10^−8^, in 0·1% (v/v) peptone water (Dilucups; LabRobot Products AB, Stenungsund, Sweden) was prepared and 0·1 ml aliquots from 10^−5^–10^−7^ dilutions were plated on 5% horse blood agar plates (SVA, 341180) for viable count and incubated for 48 ± 4 h at 41·5 ± 1°C in a microaerophilic atmosphere generated by the Anoxomat system. A 60‐l volume of autoclaved tap water was split between 6 × 10‐l containers and the first container was spiked with 1 ml from dilution 10^−6^, 10^−7^, or 10^−8^ of the *Campylobacter* cultures and mixed well by shaking. The concentration of *Campylobacter* in the dilutions was determined by viable count. The concentration was therefore not known at the time for spiking. However, previous experience was used to decide which dilutions to use in the spiking experiments. The intended spiking level was <100 CFU 60 l^−1^, aiming for isolation of *Campylobacter* from two out of three spiking levels. However, since it was difficult to assure the exact spiking levels, each spiking level is presented as a range (Table 1).

In total, 10 experiments were carried out with three different dilutions of the *Campylobacter* cultures in each of the 10 experiments. The water was pumped through filters at a rate of 2900 ml min^−1^, beginning with the container that had been spiked, followed by the other five containers. The time to process 60 l artificially contaminated water was approximately 25 min. The spiked container was rinsed thoroughly several times with water from the second container. Filtration was started with the sample spiked with the lowest concentration and ended with the sample spiked with the highest concentration, using a new filter for each sample. After filtration, the concentrated material in the filters was eluted through a so‐called ‘backflush’ system (Hill *et al.,* 2005; Smith and Hill, 2009), and 500 ml of elution buffer (phosphate‐buffered saline (PBS) supplemented with 0·01% Tween 80 (Merck KGaA8.22187, Darmstadt, Germany), 0·01% sodium polyphosphate (Merck KGaA 1.06529) and 0·001% Antifoam Y‐30 Emulsion (Merck KGaA A6457) was pumped through the system at a rate of 650 ml min^−1^. The elution buffer was pumped in through the permeate port at the top and the eluate was collected from the output port at the bottom. The eluate was further analysed for detection of *Campylobacter* according to ISO 10272‐1:2017.

### Sampling and filtration of water samples at farm level

A field study was conducted to test DEUF under field conditions. Water was sampled and filtered at farm level from five different broiler chicken producers known to deliver broiler chickens with *Campylobacter* to slaughter, according to the Swedish *Campylobacter* programme (Hansson *et al.,* 2007; 2010). The incoming water at the farms was either from a dug well or bore well. Big differences were noticed in the cleaning of water pipes during the empty period between two flocks among the different broiler chicken producers and between different flocks from the same producer. On these farms, water was sampled from different sources: a tap in an anteroom at the entrance to the broiler chicken houses and water pipes inside the broiler chicken houses. When biofilm was noticed as discoloured water or slime inside the water pipes, it was removed by increasing and decreasing the pressure of water and air in the water pipes and then collecting 50–60 l of water. In addition, surface water from two different ponds near two of the broiler chicken farms was analysed. A volume of 50–60 l water was collected in new 10‐l plastic buckets and pumped into the hollow fibre filter (REXEED‐25A) with a Cole‐Parmer Masterflex L/S peristaltic pump (model 7528‐10). The maximum pumping rate was 600 ml min^−1^ and the pressure was not allowed to exceed 0·6 bar. Filtered water exited the ultrafilter through the permeate port at the top and was allowed to drain into the sewer after filtration. After filtration at farm level, the filters were transported at 4–10°C to the laboratory, where elution and further bacteriological analysis were performed.

### Bacteriological analyses

The occurrence of *Campylobacter* spp. was analysed according to ISO 10272 part 1 (2017), with some modifications. The eluate (600–700 ml) from the filters was collected in a glass bottle and mixed with double‐concentrated Bolton broth (Oxoid CM0983, Broth Selective Supplement Oxoid SR0208E), with all ingredients except water at twice the concentration in ordinary Bolton broth (ISO, 2017). The eluate in the Bolton broth was enriched and cultured according to ISO 10272 part 1 at 37·0 ± 1°C for 4–6 h in a microaerophilic atmosphere generated by Anoxomat or CampyGen (Oxoid) and then at 41·5 ± 1°C for 44 ± 4 h with a head space of 2 cm in the bottle. After incubation, 1 ml of the enriched eluate was cultured on a mCCDA plate (Oxoid CM0739) measuring 140 mm in diameter, or on three mCCDA plates measuring 90 mm in diameter (Oxoid CM0739). All plates were incubated at 41·5 ± 1°C for 48 ± 4 h in anaerobic jars in a microaerophilic atmosphere generated by Anoxomat or CampyGen. Suspected *Campylobacter* colonies were inspected for characteristic morphology and motility using a phase contrast microscope.

Suspected *Campylobacter* colonies isolated from water sampled at farm level were confirmed and identified to species level by matrix‐assisted laser desorption/ionization time‐of‐flight mass spectrometry (MALDI‐TOF) using a Microflex LT mass spectrometer (Bruker Daltonics, Billerica, MA, USA).

### Characterization of *Campylobacter* isolates using MLST

Multi‐locus sequence types (MLST) of the isolates were determined according to Dingle *et al. *(2001) and the pubMLST database (https://pubMLST.org/campylobacter) (Jolley *et al.,* 2018).

### Statistical analysis

The probability of detection (POD) and limit of detection (LOD) were calculated using a complementary log–log model as described in Wilrich and Wilrich (2009). The calculations were performed using the PODLOD_ver9.xls spread sheet available from www.wiwiss.fu-berlin.de/fachbereich/vwl/iso/ehemalige/wilrich/index.html.

## Results

### Artificially contaminated water

Based on the results of viable counts, the estimated spiking level of *Campylobacter* per 60‐l water sample ranged from 0·1 to 2000 CFU. All strains of *Campylobacter* tested, i.e. the reference strains and strains previously isolated collected from broiler chickens, water pipes and water ponds, were detectable by DEUF at concentrations above 10 CFU 60 l^−1^ water. Furthermore, some strains (ST‐403, ST‐7516 and ST‐7809) were detectable at concentrations below 10 CFU 60 l^−1^ water (Table 1). The results of the PODLOD calculations based on the data for all strains combined show that the 95% limit of detection (LOD_95_) is 15 CFU 60 l^−1^ with the lower confidence limit 6·6 CFU 60 l^−1^ and the upper confidence limit 33 CFU 60 l^−1^ (Table 2).

### Naturally contaminated water

*Campylobacter* spp. were detected in four out of seven water samples from ponds. Three of these isolates (CA 79, CA 296 and CA 358) were identified as *C. jejuni*, while one was identified as *C. coli.* The filter used in the processing of one of the water samples from the ponds was clogged due to high turbidity. This resulted in that only 20 l could be filtered, despite that *Campylobacter* spp. were isolated from that sample. Two of the pond water samples in which *Campylobacter* spp. were detected were collected in July, the other two in December and March. In one sample from the water ponds, more than four different sequence types were identified, ST‐45, ST‐9198, ST‐693 and ST‐3137. Another pond sample contained ST‐7809 and a third sample ST‐583. Two different ST‐types (ST‐9832 and ST‐9833) were identified in the sample where *C. coli* was isolated from. *Campylobacter jejuni* was detected in three out of 24 samples collected from water pipes inside the broiler chicken houses on two different farms. On farms where *Campylobacter* spp. were isolated from the water pipes, the broiler chickens in the current flocks were carriers of *Campylobacter* and the water was heavily discoloured, with a texture and colour of coffee sump (Fig. 1). *Campylobacter jejuni* ST‐257 was isolated from two different water pipes from the same broiler chicken producer. Whereas the third *C. jejuni* isolated from the water from the water pipe sampled from another farm was ST‐21. *Campylobacter* spp. were not detected in any of the 22 samples of tap water taken in an anteroom at the entrance to the broiler chicken houses (Table 3).

**Table 3:** *Campylobacter* detection by dead‐end ultrafiltration in 50–60 l water samples collected on broiler farms frequently delivering broilers with *Campylobacter* to slaughter, according to the Swedish *Campylobacter* program

	No growth of *Campylobacter*	Growth of *C. jejuni*	Growth of *C. coli*	Total number of samples
Tap water at the entrance to the broiler chicken house	22			22
Water from pipes inside the broiler chicken house	21	3		24
Surface water from a pond near the broiler chicken house	3	3	1	7
Total number of samples	46	6	1	53

## Discussion

The results obtained demonstrate that DEUF is a reliable and sensitive technique for processing and concentrating large volumes of water to recover low numbers of *Campylobacter*. *Campylobacter* spp. were isolated from all samples spiked with at least 10 CFU 60 l^−1^ water. The variety of strains used in the spiking experiments, both in source and genetic set‐up, showed that DEUF can be used for detection of different strains of *Campylobacter* spp. In some samples, *Campylobacter* could even be detected in concentrations below 10 CFU 60 l^−1^ water. However, the variation in detection results at low spiking levels indicates that a level of around 10 CFU 60 l^−1^ is close to the detection limit of the method, which was also shown by the statistical analysis where LOD_95_ was 15 CFU 60 l^−1^ with the confidence interval 6·6–33 CFU 60 l^−1^. Although DEUF has previously been used for *Campylobacter* in water (Li *et al.,* 2016), to our knowledge we are the first to report the detection limit of DEUF for *Campylobacter* in water. The detection limit identified in this study agrees with previous work on other organisms, which has demonstrated that viable spores of *Bacillus artrophaeus* at levels of <10 CFU l^−1^ can be concentrated and recovered from >100 l tap water (Kearns *et al.,* 2008) and that high recovery rates (>95%) can be obtained for *Escherichia coli* and human polyomavirus using DEUF (Li *et al.,* 2016). Also, Humrighouse *et al. *(2015) determined the method detection limit for a tangential flow ultrafiltration‐based automated waterborne pathogen concentrator using the same filter type as in this study. Their experimental data showed a method detection limit for *Bacillus anthracis* of approximately 6 CFU 100^−l^ with the confidence interval 4·8–8·4 CFU 100^−l^.

There are several factors that can influence detection of *Campylobacter* in water, despite using sensitive methods. One is that *Campylobacter* can be in a viable but non‐culturable form due to environmental stress (Rollins and Colwell, 1986). Another is that if there is an uneven distribution of *Campylobacter* in the water or if the contamination level is below the detection limit of the method, the results of the analysis may be false‐negative. This can result in incorrect assessment of the *Campylobacter* risk posed by drinking and environmental waters.

Compared with other food‐borne pathogens, *Campylobacter* spp. are unusually sensitive to different types of environmental stress, such as desiccation (Fernandez *et al.,* 1985), oxidative stress, osmotic stress (Doyle and Roman, 1982) and high temperatures (Park, 2002). The sensitivity of culture‐based detection methods is therefore improved by analysis of larger volumes of water. In fact, it has been shown in practice that analysis of larger water volumes improves the POD for *Campylobacter* (Hanninen *et al.,* 2003; Pitkänen *et al.,* 2009). The concentrations of *Campylobacter* in different water sources are highly variable, with reported levels from <1 CFU l^−1^ in drinking water to up to 4 600 000 CFU l^−1^ in environmental water (Savill *et al.,* 2001; Diergaardt *et al.,* 2004; Vereen *et al.,* 2007; St‐Pierre *et al.,* 2009; Hellein *et al.,* 2011; Hokajarvi *et al.,* 2013; Banting *et al.,* 2016). However, these levels have been determined using different types of quantitative methods, such as direct plating, most‐probable‐number culture enrichment and quantitative PCR, which impedes direct comparisons between different studies. However, in most cases, the reported level of *Campylobacter* in water samples is low, less than 10 CFU l^−1^ (Savill *et al.,* 2001; Diergaardt *et al.,* 2004; St‐Pierre *et al.,* 2009; Banting *et al.,* 2016).

One limitation to detection of *Campylobacter* in water using the ultrafiltration technique is the turbidity of the water, as highly turbid water can cause clogging of the filter (Olszewski *et al.,* 2005). In this study, *Campylobacter* spp. were isolated from those water samples with the highest turbidity with the tendency of clogging the filter. This could be due to the ability of *Campylobacter* to adapt to survival in the environment, exhibiting aerotolerance and interact with other micro‐organisms. It is well known that *C. jejuni* could survive in biofilms in water systems since they can protect constituent micro‐organisms from environmental stress (Trachoo *et al.* 2015; Bronowski *et al*. 2014). *Campylobacter* could also be present within amoebic vacuoles in water, and it could survive for longer within these vacuoles than as extracellular bacteria. *Campylobacter jejuni* can infect protozoan *Acanthamoeba polyphaga* cells *in vitro* and the protozoa can act as a reservoir and vector for *Campylobacter* (Axelsson‐Olsson *et al.,* 2005). The DEUF method is a modification of hollow fibre ultrafiltration techniques, which generally employ a tangential flow approach that requires comprehensive operator training (Smith and Hill, 2009). DEUF is an alternative approach to tangential‐flow hollow fibre ultrafiltration that can be readily employed under field conditions to recover microbes from water. In comparisons of different ultrafilters, the sample processing rate has been found to be higher for REXEED 25S filters (2 l min^−1^) used in DEUF than for some recirculating (tangential‐flow) hollow fibre ultrafiltration methods (Hill *et al.,* 2007). The DEUF method can be efficient for sample collection and recovery of microbes present in 100‐l water samples of low to moderate turbidity (Smith and Hill, 2009). Another advantage of DEUF is that it is less likely to clog than other ultrafiltration methods using filters with lower surface area, which is an important factor for the performance of this technique.

While many studies have examined transmission routes for *Campylobacter* to broiler chickens, there are still knowledge gaps concerning how to detect and avoid transmission of *Campylobacter* from the environment and water (Hansson *et al.,* 2018). Among the water samples taken from broiler chicken farms in the present study, *Campylobacter* spp. were most frequently detected in water taken from ponds close to the broiler chicken houses. This was expected, as the ponds in question are frequently visited by wild animals, e.g. wild boar, and wild birds, e.g. mallards. Wild boar and mallards are both known to be carriers of *Campylobacter* (Cummings *et al.,* 2018). The mechanisms behind host specificity for bacterial pathogens are multifactorial and include ingestion, replication in the host and competition with the surrounding microbiota (Baumler and Fang, 2013). *Campylobacter* spp. were detected in three of the 24 samples taken from water pipes inside the broiler chicken houses in the present study, but not in any of the 22 samples of tap water taken in an anteroom at the entrance to the broiler chicken houses. One possible explanation is that *Campylobacter* survived in biofilm in the water pipes inside the broiler chicken houses, due to inadequate cleaning and disinfection. Biofilm consists of population(s) of bacteria, which adhere to a surface and to each other and are enclosed in a network of biopolymers. Bacteria in biofilm are known to be more resistant to detergents and phagocytosis than planktonic bacteria (Hanning *et al.,* 2008; Reuter *et al.,* 2010; Teh *et al.,* 2010; Bronowski *et al.,* 2014; Turonova *et al.,* 2015). This may pose a risk of subsequent broiler chicken flocks being exposed to *Campylobacter* (Teh *et al.,* 2016). One challenge to detecting *Campylobacter* in biofilm inside water pipes is that the biofilm can adhere strongly to the pipe interior and form several layers. The farm where *C. jejuni* ST‐257 was isolated from the water pipes has previously, during four different rotations, delivered chicken with *C. jejuni* ST‐257 to slaughter within the Swedish Campylobacter programme (Hansson *et al.* 2007). After cleaning of the water pipes *C. jejuni* ST‐257 has not been isolated from broiler chickens from that producer. This result together with the discoloured water from the water pipes indicates that there was *Campylobacter*‐containing biofilm present on the inside of the pipes and that it was removed from the pipes due to the varying water pressure and air applied during sampling.

In conclusion, in epidemiological studies of *Campylobacter* in water, sensitive methods are needed for its detection in water samples, which often contain low levels of *Campylobacter*. The results of the present study indicate that DEUF can be an effective technique for rapid sample collection and efficient recovery of low numbers of *Campylobacter* in water volumes of more than 50 l. An advantage of this technique is that untrained personnel can perform the analysis under field conditions, which makes it useful for monitoring the presence of micro‐organisms in both tap water and surface water. Another advantage of the technique is that it increases the probability of detecting *Campylobacter* in water, as it makes it possible to analyse large volumes of water.

## Conflict of interest

There are no conflicts of interest to declare.
